# The Brazilian version of the Health-Related Quality of Life Questionnaire for Nausea and Vomiting of Pregnancy: translation, cross-cultural adaptation and reliability – an observational cross-sectional study

**DOI:** 10.1590/1516-3180.2020.0430.R1.08122020

**Published:** 2021-04-05

**Authors:** Adriana Piccini, Amanda Tulha, Sílvia Lanziotti Azevedo da Silva, Luciana de Barros Cavalcanti Michelutti, Leonardo César Carvalho, Simone Botelho

**Affiliations:** I MSc. Physiotherapist, Postgraduate Program on Rehabilitation Sciences, Motor Science Institute, Federal University of Alfenas (UNIFAL-MG), Alfenas (MG), Brazil.; II BSPT. Physiotherapist, Physiotherapy Course, Motor Science Institute, Federal University of Alfenas (UNIFAL-MG), Alfenas (MG), Brazil.; III PhD. Professor, Faculty of Medicine/Department of Collective Health, Federal University of Juiz de Fora (UFJF), Juiz de Fora (MG), Brazil; Professor, Postgraduate Program on Rehabilitation Sciences, Motor Science Institute, Federal University of Alfenas (UNIFAL-MG), Alfenas (MG), Brazil.; IV BSc. Professor, School of Medicine, Federal University of Alfenas (UNIFAL-MG), Alfenas (MG), Brazil; Master's Student, Postgraduate Program on Rehabilitation Sciences, Motor Science Institute, Federal University of Alfenas (UNIFAL-MG), Alfenas (MG), Brazil.; V PhD. Physiotherapist and Professor, Postgraduate Program on Rehabilitation Sciences, Motor Science Institute, Federal University of Alfenas (UNIFAL-MG), Alfenas (MG), Brazil.; VI PhD. Physiotherapist, Professor and Coordinator, Postgraduate Program on Rehabilitation Sciences, Motor Science Institute, Federal University of Alfenas (UNIFAL-MG), Alfenas (MG), Brazil; Professor and Researcher, Postgraduate Program on Surgical Science, School of Medical Sciences, State University of Campinas (UNICAMP), Campinas (SP), Brazil.

**Keywords:** Nausea, Rehabilitation, Surveys and questionnaires, Transcultural study, Vomiting, Emesis, Physiotherapy, Pregnancy, Pregnant, Quality of life, Validation

## Abstract

**BACKGROUND:**

The Health-Related Quality of Life Questionnaire for Nausea and Vomiting of Pregnancy (NVPQOL) is a validated questionnaire assessing quality of life among pregnant women with nausea and vomiting.

**OBJECTIVE:**

To translate, cross-culturally adapt and evaluate the reliability of the NVPQOL.

**DESIGN AND SETTING:**

Observational cross-sectional study developed in a public university in Brazil.

**METHODS:**

The translation, synthesis of translations, back-translation, expert committee, pre-testing and validation were carried out, resulting in a Portuguese-language version. The internal consistency, intra-rater and test-retest reliability and correlation between the total score of the Portuguese-language version of the NVPQOL and the domains of the World Health Organization Quality of Life-bref questionnaire were considered in the data analysis.

**RESULTS:**

The instrument went through the process with testing on 104 pregnant women. Strong internal consistency (Cronbach's α: 0.95), strong intra-rater and test-retest reliability (P < 0.0; intraclass correlation coefficient: 0.89; confidence interval: 0.791-0.945) and strong correlation between the total score of the Portuguese-language version of the NVPQOL and the physical health domain of the World Health Organization Quality of Life-bref questionnaire (P < 0.01; R = −0.8) were observed.

**CONCLUSION:**

The NVPQOL was translated, cross-culturally adapted and validated for the Portuguese language with satisfactory psychometric properties for assessing quality of life, especially in relation to physical health, among pregnant women with symptoms of nausea and vomiting in the first trimester of pregnancy.

## INTRODUCTION

Nausea is defined as an uncomfortable sensation that is associated with the urge to vomit, while vomiting is characterized by oral expulsion of gastric contents.^1^ These symptoms are common during the first gestational trimester, affecting 70-80% of pregnant women, and their cause is still uncertain.^2^^,^^3^ They are responsible for physical and emotional impairment, which tends to trigger social isolation and compromise quality of life, given that, according to Hizli et al.,^4^ 8 to 12% of pregnant women with these symptoms isolate themselves socially. Pregnant women's common complaints include uncomfortable symptoms of physical discomfort that prevent them from successfully performing their daily activities. These involve negative feelings about pregnancy, which can give rise to emotional conflicts in these women's relationships with the baby and family.

These symptoms are more prevalent among young, multiparous and multiple gestation pregnant women.^5^ They tend to soften after the 22^nd^ gestational week,^6^ although in some women they may continue until the end of the gestation,^6^ thus characterizing pregnancy hyperemesis. In such cases, the symptoms of nausea and vomiting of pregnancy are persistent, excessively compromising and untreatable. They may lead to dehydration, ketosis, weight loss and electrolyte and nutritional disorders.^4^^,^^7^^,^^8^

Investigating the frequency of these symptoms and their impact on women's quality of life can help professionals to understand this condition, in order to minimize the impact of nausea and vomiting on this very special phase of women's lives. For this purpose, Lacasse et al.^9^ created the Modified Pregnancy – Unique Quantification of Emesis and Nausea questionnaire, which investigates the presence and severity of nausea and vomiting among pregnant women in the first trimester of pregnancy. Additionally, the same authors validated the Health-Related Quality of Life Questionnaire for Nausea and Vomiting of Pregnancy,^9^ a questionnaire created by Magee et al.,^10^ in order to measure the impact of specific nausea and vomiting of pregnancy on quality of life in the first trimester of pregnancy.

Currently, in Brazil, there is no instrument for measuring the impact of nausea and vomiting of pregnancy on pregnant women's quality of life, which therefore justified conducting the present study. Thus, we proposed to undertake the translation, cross-cultural adaptation and evaluation of the reliability of the Health-Related Quality of Life Questionnaire for Nausea and Vomiting of Pregnancy, in order to provide the Brazilian population with a reliable instrument for assessing the quality of life of pregnant women with symptoms of nausea and/or vomiting.

## OBJECTIVE

To translate, cross-culturally adapt and evaluate the reliability of the Health-Related Quality of Life Questionnaire for Nausea and Vomiting of Pregnancy.

## METHODS

### Study type

This was an observational cross-sectional study.

### Description of the Health-Related Quality of Life Questionnaire for Nausea and Vomiting of Pregnancy

The Health-Related Quality of Life Questionnaire for Nausea and Vomiting of Pregnancy is a self-administered questionnaire that was developed by Magee et al.^10^ and validated by Lacasse et al.^9^ in 2008. This instrument assesses quality of life among pregnant women with symptoms of nausea and vomiting of pregnancy and was originally validated among pregnant women in their first trimester of pregnancy. It is composed of 30 questions covering four main domains: physical symptoms and aggravating factors, fatigue, emotions and limitations.^10^ For each question in the questionnaire, a range of possible responses is provided using a seven-point Likert scale, on which 1 represents “none of the time” and 7 represents “all of the time”. The sum of the scores from these questions results in a total score ranging from 30 to 210. The lower the score is, the higher the quality of life is.^9^

### Ethical issues

In order to begin the study, authorization was sought from the author Dr. Laura Magee, which was granted. Furthermore, approval was given by the Institutional Review Board of the study university, in accordance with the ethical and legal precepts (Institutional Review Board approval CAAE: 81392017.1.0000.5142; approval number: 2.543.785; approval date: March 14, 2018). All the study participants signed an informed consent statement.

### Translation, cross-cultural adaptation and content validation processes of the Health-Related Quality of Life Questionnaire for Nausea and Vomiting of Pregnancy

The stages proposed by Beaton et al.^11^ were used as a reference framework for developing the translation and cross-cultural adaptation stages of the Health-Related Quality of Life Questionnaire for Nausea and Vomiting of Pregnancy, in accordance with the COSMIN recommendations.^12^

#### Stage I: Initial translation

The original Health-Related Quality of Life Questionnaire for Nausea and Vomiting of Pregnancy went through the process of translation into Portuguese by two independent bilingual translators, who were Brazilians: one of them was lay and the other was a specialist in the field. Thus, two translations (T1 and T2) containing the instructions, questions and responses to the questionnaire were generated.

#### Stage II: Synthesis

The two versions generated in stage I (T1 and T2) were synthesized into a common version (T12) and all divergences were reported and agreed by the researchers responsible for this study.

#### Stage III: Back translation

The synthesized version (T12) was back-translated by two independent translators, who were native speakers of the English language and not healthcare professionals. Thus, two back-translated versions (BT1 and BT2) were generated. These presented content that was similar to that of the original version, thus ensuring consistency of the translation.

#### Stage IV: Expert committee

In order to verify equivalence, an expert committee composed of 12 healthcare professionals (seven physiotherapists, three nurses and two physicians) evaluated the translated versions (T1, T2, T12, BT1 and BT2) and approved the pre-final version of the questionnaire (Health-Related Quality of Life Questionnaire for Nausea and Vomiting of Pregnancy_pre-final_version).

#### Stage V: Pre-testing

The Health-Related Quality of Life Questionnaire for Nausea and Vomiting of Pregnancy_pre-final_version was applied to 104 pregnant women who had been selected from among attendees at a doctor's office and family health program in Alfenas (MG), Brazil. The aim in doing this was to identify any adaptations that might be necessary, ensure that the target population understood the questions and test the semantic, idiomatic, experimental and conceptual equivalence. This sample was defined for convenience. Thus, after the pretest, the final version was generated (Health-Related Quality of Life Questionnaire for Nausea and Vomiting of Pregnancy_Portuguese), as shown in **Annex 1**.

### Evaluation of the reliability of the Health-Related Quality of Life Questionnaire for Nausea and Vomiting of Pregnancy_Portuguese

After the translation, cross-cultural adaptation and content validation processes had ended, the reliability of the instrument was ascertained among another 36 pregnant women. These subjects were recruited from among the attendees at doctors’ offices and the family health program of the municipality of Alfenas (MG), Brazil. These women attended two face-to-face interviews conducted by the same researcher, who had been trained and qualified to maintain an interview standard. There was a seven-day interval between the interviews.^13^

At both interviews, the subjects gave responses to two instruments: the Health-Related Quality of Life Questionnaire for Nausea and Vomiting of Pregnancy_Portuguese and the World Health Organization Quality of Life-bref questionnaire. The World Health Organization Quality of Life-bref questionnaire had previously been validated by Fleck et al.^14^ It is composed of 26 questions, including two general questions and 24 questions divided into four domains: physical, psychological, social relations and environment.^15^ This questionnaire was chosen for comparison purposes considering that no other gold standard that assessed the quality of life of individuals with symptoms of nausea and vomiting was found.

The inclusion criteria were that the participants needed to be over 18 years of age, with a pregnancy of gestational age less than 16 weeks. Women presenting neurological abnormalities, disorders and/or cognitive limitations that precluded participation in the study were excluded. Women who did not participate fully in the proposed activities, i.e. attendance at both interviews, were considered to have discontinued their participation.

### Data analysis

The data analysis was carried out by two researchers and the following tests were performed: Cronbach's alpha coefficient test for internal consistency analysis (considering the first interview);^16^ intraclass correlation coefficient (ICC) (model 2.1; two-way random; single measurement) for test-retest reliability analysis (considering instrument total scores at the first and second interviews);^16^^,^^17^ Shapiro-Wilk test followed by the Spearman or Pearson correlation test to investigate the relationship between the total score of the Health-Related Quality of Life Questionnaire for Nausea and Vomiting of Pregnancy_Portuguese and the domain scores of the World Health Organization Quality of Life-bref questionnaire.

The Microsoft Excel 2010 software, version 14.4.9 (Microsoft Corporation, Redmond, WA, United States) and the R program software, version 3.3.3 (https://www.r-project.org/, Vienna, Austria) were used for data analysis. Coefficients below 0.5 were considered to present “poor reliability”; coefficients between 0.5 and 0.75, “moderate reliability”; and coefficients above 0.75, “strong reliability”.^16^ In all the analyses, a 95% confidence interval (CI) was used.

No inter-rater analysis was performed, given the self-applicable nature of the instrument, which does not require any intervention from the evaluator.

The Survio platform was used to make the questionnaires available and to collect answers in the different stages of the translation and expert committee analyses.

## RESULTS

The whole process involved 140 pregnant women, who participated either in the pretesting process (n = 104) or in the reliability process (n = 36). **Table 1** presents the study population for the process of cross-cultural adaptation and evaluation of the reliability of the Health-Related Quality of Life Questionnaire for Nausea and Vomiting of Pregnancy_Portuguese.

**Table 1:** Demographic and clinical characteristics of the study population

Demographic data		Pretesting (n = 104)	Reliability (n = 36)
**Age**, years (standard deviation)^a^		26.5 (± 5.6)	27.1 (± 5.5)
**Education level**, n (%)^b^			
	Elementary school	16 (15.4)	6 (16.7)
	High school	66 (63.5)	12 (33.3)
	University/college	22 (21.1)	18 (50)
**Household income**, n (%)^b^			
	1-2 minimum monthly wages	74 (71.2)	22 (61.1)
	3-4 minimum monthly wages	23 (22.1)	6 (16.7)
	> 4 minimum monthly wages	7 (6.7)	8 (22.2)
**Clinical data**		**Pretesting** **(n = 104)**	**Reliability** **(n = 36)**
**Gestational age**, weeks (standard deviation)^a^		22.3 (± 10.4)	11.36 (± 3.67)
**Nausea and vomiting presence**, n (%)^b^			
	No	56 (53.8)	11 (30.6)
	Yes	48 (46.2)	25 (69.4)
**Antiemetic use**, n (%)^b^			
	No	91 (87.5)	23 (63.9)
	Yes	13 (12.5)	13 (36.1)

a Mean and standard deviation (±)

b Absolute number (n) and relative frequency (%).

### Translation and cross-cultural adaptation

**Table 2** presents the adaptations made during the process of translation and cross-cultural adaptation, through conformity analysis between the original and translated versions of the instrument.

**Table 2:** Process of translation and cross-cultural adaptation of the items of the Health-Related Quality of Life Questionnaire for Nausea and Vomiting of Pregnancy_Portuguese

Original version	T1	T2	T12	Pre-final version	Final version
Over the past week, how much have you been experiencing… (Please fill in one circle on the scale for each symptom)	*Durante a última semana, quantas vezes você teve os sintomas abaixo… (Colocar às vezes dentro do círculo para cada sintoma)*	*No período de uma semana, quantas vezes você sentiu… (Marque um círculo para cada sintoma)*	*Durante a última semana, quantas vezes você teve os sintomas abaixo… (Marque um círculo para cada sintoma)*	*Durante a última semana, quantas vezes você* **apresentou os seguintes sintomas… (Por favor, marque um círculo na escala para cada sintoma)**	*---*
None of the time	*Nenhuma vez*	*Nunca*	*Nenhuma vez*	*---*	*---*
All of the time	*O tempo todo*	*Sempre*	*O tempo todo*	*---*	*---*
Nausea	*Náusea*	*Ânsia*	*Náusea*	**Enjoo**	*---*
Feeling sick to your stomach	*Estômago enjoado*	*Ânsia estomacal*	*Estômago enjoado*	**Dor no estômago**	*---*
Vomiting	*Vomitar*	*Vomitando*	*Vomitar*	*Vomitar*	**Vômito**
Dry-heaves (vomiting without bringing anything up)	*Ânsia de vômito*	*Ânsia de vômito*	*Ânsia de vômito*	*---*	*---*
Poor appetite	*Sem apetite*	*Pouco apetite*	*Pouco apetite*	**Perda de apetite**	*---*
Symptoms being worse in the evening	*Sintomas pioram à tarde*	*Sintomas pioram à tarde*	*Sintomas pioram à tarde*	**Piora dos sintomas no período da tarde**	*---*
Not eating for longer than you would like	*Não se alimentar mais do que queria*	*Não comer pelo tempo que quiser*	*Não comer o quanto gostaria*	*Não* **posso** *comer o quanto* **desejaria**	*---*
Feeling worse when exposed to certain smells	*Sentir pior quando exposta a alguns cheiros*	*Sentir mal com cheiro forte*	*Se sente pior quando exposta a certos cheiros*	*Se sente pior quando* **sente** *certos cheiros*	*---*
Feeling worse when exposed to certain foods	*Sentir pior quando exposta a certos alimentos*	*Sentir mal com certas comidas*	*Se sente pior quando exposta a certas comidas*	*Se sente pior quando* **come** *certas comidas*	*---*
Fatigue	*Cansada*	*Cansada*	*Cansada*	**Fadiga**	*---*
Feeling worn-out and a loss of energy	*Sentir cansada e sem energia*	*Sentir esgotada e com pouca energia*	*Se sente esgotada e sem energia*	*---*	*---*
Feeling exhausted	*Se sentir exausta*	*Sentir exausta*	*Se sente exausta*	*---*	*---*
Feeling tired	*Se sentir cansada*	*Sentir cansada*	*Se sente cansada*	*---*	*---*
Feeling emotional	*Se sentir emotiva*	*Sentir emotiva*	*Se sente emotiva*	*---*	*---*
Being less interested in sex	*Sentir menos interesse em sexo*	*Ter menos interesse por sexo*	*Sente menos interessada em sexo*	*Sente menos* **interesse** *em sexo*	*---*
Feeling downhearted, blue, sad, unhappy, depressed, gloomy	*Sentir pra desanimada, triste, infeliz, deprimida e sem vontade*	*Sentir para baixo, triste, infeliz, deprimida sem vida*	*Se sente para baixo, desanimada, triste, infeliz, deprimida e sem vontade*	*---*	*---*
Feeling frustrated	*Se sentir frustrada*	*Sentir frustrada*	*Se sente frustrada*	*---*	*---*
Feeling fed up with being sick	*Ficar brava por estar doente*	*Sentir raiva de estar doente*	*Se sente brava por estar doente*	*Se sente* **com raiva** *por estar* **enjoada**	*---*
Not feeling that your symptoms are all part of normal pregnancy	*Não perceber que os sintomas são partes da gravidez*	*Achar que os sintomas não fazem parte da gravidez*	*Não sente que todos os sintomas são de uma gravidez normal*	*Sente que todos seus sintomas não* **correspondem** *a uma gravidez normal*	*Sente que seus sintomas* **não são de** *uma gravidez normal*
Feeling that you can't enjoy your pregnancy	*Sentir que não irá aproveitar a gravidez*	*Sentir que não pode apreciar a gravidez*	*Sente que você não pode aproveitar a gravidez*	*Sente que você não pode* **apreciar** *a gravidez*	*---*
That everything is an effort	*Tudo é um esforço*	*Que tudo é um esforço*	*Que tudo é um esforço*	*Tudo é um esforço*	*---*
Feeling like you have accomplished less than you would like	*Sentir que fez menos do que poderia*	*Sentir que fez menos do que poderia*	*Sente que você fez menos do que gostaria*	*Sente que você* **faz** *menos do que* **gostaria**	*---*
That it takes longer to get things done than usual	*Demorar mais para fazer algo era comum*	*Que demora mais para fazer algo*	*Demora mais para fazer algo do que o comum*	**Leva mais tempo** *para fazer* **as coisas do que normalmente**	*---*
Difficulty performing your work and activities	*Dificuldade para realizar um trabalho ou uma atividade*	*Dificuldade em completar alguma atividade*	*Dificuldade em realizar seu trabalho e atividades*	*---*	*---*
Difficulty maintaining your normal social activities	*Dificuldade para manter atividades sociais*	*Dificuldade de manter uma vida social*	*Dificuldade em manter suas atividades sociais*	*---*	*---*
Relying on your partner for doing things that you would normally	*Depender de seu companheiro para fazer as coisas que antes fazia normalmente*	*Depender de seu parceiro para fazer algo que fazia sozinha*	*Depende do seu parceiro para fazer coisas que você faria normalmente*	*---*	*---*
Difficulty looking after your home	*Dificuldade de cuidar da casa*	*Dificuldade de fazer trabalhos domésticos*	*Dificuldade em cuidar de sua casa*	*---*	*---*
Difficulty shopping for food	*Dificuldade em fazer compras*	*Dificuldade em fazer compras*	*Dificuldade em fazer compras*	*---*	*---*
Difficulty preparing or cooking meals	*Dificuldade em cozinhar*	*Dificuldade em cozinhar*	*Dificuldade em cozinhar*	*---*	*---*
Cutting down on amount of time you spend at work or other activities	*Diminuir o tempo de trabalho ou das tarefas normais*	*Diminuir o tempo de atividades*	*Redução do tempo de trabalho ou outras atividades*	*---*	*---*

This table presents the initial translation version, made by two Portuguese native speakers (T1 and T2); the synthesis version (T12); the pre-final version (Health-Related Quality of Life Questionnaire for Nausea and Vomiting of Pregnancy_pre-final_version) that was applied during the pre-test; and the final version named the Health-Related Quality of Life Questionnaire for Nausea and Vomiting of Pregnancy_Portuguese.

--- = unchanged.

### Instrument validation

#### Internal consistency

The Health-Related Quality of Life Questionnaire for Nausea and Vomiting of Pregnancy_Portuguese showed strong internal consistency (Cronbach's α coefficient = 0.95).

#### Instrument reliability

A strong correlation was observed in the test-retest analysis on the Health-Related Quality of Life Questionnaire for Nausea and Vomiting of Pregnancy_Portuguese (ICC = 0.89; 95% CI: 0.791-0.945; P < 0.0).

#### Instrument correlation

The total score of the Health-Related Quality of Life Questionnaire for Nausea and Vomiting of Pregnancy_Portuguese was correlated with the score for each domain of the World Health Organization Quality of Life-bref questionnaire. The presence of nausea and vomiting symptoms (high score in the Health-Related Quality of Life Questionnaire for Nausea and Vomiting of Pregnancy_Portuguese) was correlated with low quality of life scores, especially with regard to physical function (low domain scores in the World Health Organization Quality of Life-bref questionnaire) (**Table 3**). Clinically, an indirect relationship was observed between the nausea and vomiting symptoms and the physical health domain of the World Health Organization Quality of Life-bref questionnaire. This indicated that the greater the nausea and vomiting symptoms were, the greater the impairment of the quality of life of the pregnant women in the study also was.

**Table 3:** Reproducibility and instrument correlation analysis on the Health-Related Quality of Life Questionnaire for Nausea and Vomiting of Pregnancy_Portuguese

Domains of the World Health Organization Quality of Life-bref questionnaire	Health-Related Quality of Life Questionnaire for Nausea and Vomiting of Pregnancy_Portuguese (P-value)	Correlation coefficient (R)
Overall perception of quality of life	< 0.01^a^*	-0.55
Overall perception of general health	< 0.01^a^*	-0.55
Physical health	< 0.01^a^*	-0.80
Psychological	< 0.01^a^*	-0.68
Social relationships	< 0.01^a^*	-0.53
Environment	0.01^b^*	-0.42

a Spearman correlation test

b Pearson correlation test

* P-value < 0.05.

## DISCUSSION

### Main findings

The Health-Related Quality of Life Questionnaire for Nausea and Vomiting of Pregnancy was translated^9^^,^^10^ and cross-culturally adapted for the Portuguese language, and it presented good test-retest reliability and good correlation with the domains of the World Health Organization Quality of Life-bref questionnaire. The instrument showed satisfactory psychometric properties for assessing quality of life among pregnant women with symptoms of nausea and vomiting in the first gestational trimester.

The Portuguese-language version (Health-Related Quality of Life Questionnaire for Nausea and Vomiting of Pregnancy_Portuguese) presented strong internal consistency, strong intra-rater and test-retest reliability and strong correlation between the total score of the Health-Related Quality of Life Questionnaire for Nausea and Vomiting of Pregnancy Portuguese and the physical health domain of the World Health Organization Quality of Life-bref questionnaire. Thus, the translated questionnaire was reliable for measuring the impact of nausea and vomiting on the physical health aspect of quality of life among Brazilian pregnant women.

The Health-Related Quality of Life Questionnaire for Nausea and Vomiting of Pregnancy was originally created by Magee et al.^10^ in 2002, using a sample of 500 women with nausea and vomiting of pregnancy. The methodology gave value to potential factors that correlate nausea and vomiting of pregnancy with quality of life. The validation study among women in the first trimester of pregnancy was conducted by Lacasse et al.^9^ in 2008, using a sample of 288 pregnant women with symptoms of nausea and vomiting of pregnancy. Our study was designed in accordance with the same criteria used by Lacasse et al.,^9^ in spite of adding the test-retest and intra-rater reliability analyses.

Our study found excellent internal reliability for the complete questionnaire, thus corroborating the results from the study by Lacasse et al.^9^ In theory, reliability analyses follow the condition that the higher the test-retest reliability is, the higher the internal reliability also is, which was observed in the present study.^18^

### Clinical and research implications

Health status is a factor relating to quality of life that is one of the essential components of health-related quality of life.^19^ During the gestational period, there are important physiological changes and consequent emotional and physical changes that impact on women's health and quality of life, and these may negatively affect the mother-child binomial.

It is known that symptoms of nausea and vomiting of pregnancy affect up to 80% of women. They have greatest occurrence in the first trimester^20^ and tend to decrease after this period. This justifies investigation of such symptoms during this period, through clinical research on the impact of nausea and vomiting of pregnancy on the quality of life of pregnant women.

Our study involved 140 women during the process of cross-cultural adaptation and validation, and we found that the questionnaire presented strong statistical validity, thus demonstrating its psychometric properties with regard to women in their first trimester of pregnancy.

According to the American College of Obstetricians and Gynecologists,^21^ most cases of nausea and vomiting of pregnancy are self-limiting with a peak incidence around the 9^th^ gestational week and symptom relief around the 20^th^ gestational week. The present study was conducted with a limit on assessment and reassessment (test-retest) that was set at the 16^th^ week of gestation, as was done in the validation study conducted by Lacasse et al.,^9^ although the original study included women up to the 20^th^ week.^10^

This approach was justified not only by the fact that the prevalence is highest in the first trimester, but also because complaints that tend to persist throughout the gestational process are already characterized as *hyperemesis gravidarum* and may involve other related factors, despite lack of full knowledge of the etiology so far.^22^ In addition, the effect of memory and other characteristic symptoms of each gestational phase could influence the perception of health and consequently the quality of life in other gestational periods, such as the sensation of heartburn, which is a common characteristic of the last gestational trimester.^23^

Additionally, the emotional conflict experienced by these women, involving the mother-child relationship, needs to be considered, given that the impact of any distress after the acute phase of nausea and vomiting tends to be minimized. In this regard, reliability studies have affirmed that the time interval between measurements may influence the interpretation of the test-retest reliability.^17^ Therefore, we took care not only to always reapply the instrument after a seven-day interval, but also to ensure that the whole process was completed before reaching 16 weeks of gestation.

Improving the measurement quality of factors relating to the quality of life of pregnant women with symptoms of nausea and vomiting was one of the main contributions of the present study to clinical practice.

### Quality of life implications

A strong correlation was found between the total score of the Health-Related Quality of Life Questionnaire for Nausea and Vomiting of Pregnancy_Portuguese and the physical health domain of the World Health Organization Quality of Life-bref questionnaire. In a study by Attard et al.,^24^ it was found that pregnant women who had at least one of the symptoms of nausea, vomiting or fatigue also had significantly lower scores in assessments of quality of life specifically in the physical and mental domains, such that their health was negatively affected.

The presence of symptoms of nausea and vomiting of pregnancy is known to affect women's performance in their work and daily activities.^25^ In this regard, these symptoms seem to have a negative impact when analyzed from the point of view of the international classification of functionality,^26^ especially in terms of activity and participation. However, there are no studies linking women with nausea and vomiting of pregnancy to their functional capacity. This would be an important approach for future research, especially because this negative impact was observed by the present researchers in developing this study.

The World Health Organization Quality of Life-bref domains of overall perception of quality of life, overall perception of general health, psychological factors and social relationships showed moderate correlations with the total score of the Health-Related Quality of Life Questionnaire for Nausea and Vomiting of Pregnancy_Portuguese. This demonstrated that the symptoms of nausea and vomiting of pregnancy may also affect other matters that constitute quality of life overall. In a study by Chou et al.,^27^ it was observed that symptoms of nausea and vomiting of pregnancy were associated with depressive symptoms, which may explain the correlation with the psychological domain.

However, there are inconsistencies between different studies that may reflect differences between populations. In interpreting the results from these studies in relation to quality of life, it is necessary to consider their environmental and cultural aspects.^28^ In this regard, the present study showed a weak correlation with the symptoms of nausea and vomiting of pregnancy in the environment domain of the World Health Organization Quality of Life-bref questionnaire.

We observed that the strong correlation between the total score of the Health-Related Quality of Life Questionnaire for Nausea and Vomiting of Pregnancy_Portuguese and the physical health domain of the World Health Organization Quality of Life-bref questionnaire stood out in relation to comparisons with the general perception of quality of life and the health, psychological, social relationship and environment domains. This suggested, in interpreting the instrument, that there was a real relationship between the symptoms of nausea and vomiting of pregnancy and the physical health perceptions of pregnant women.

This finding also demonstrates the importance of thoroughness in interpreting the validation process of this study. We found greater correlations between certain domains of these two instruments, which have different scoring systems and ways of calculating them.

### Strengths, limitations and suggestions for further studies

We suggest that future research should include use of the Health-Related Quality of Life Questionnaire for Nausea and Vomiting of Pregnancy_Portuguese in other gestational periods, i.e. in the second and third gestational trimesters. However, this does not mean that the questionnaire cannot already be used to access the symptoms of nausea and vomiting of pregnancy throughout the gestational period. What we want to point out is that we were unable to identify any studies investigating the validity, and no other instruments investigating nausea and vomiting of pregnancy, at these specific stages of pregnancy so far. This might be considered a limitation of our study. Follow-up pregnant women could provide enlightenment on whether nausea and vomiting of pregnancy in the first trimester of pregnancy really is a turning point in the quality of life of these women.

Furthermore, we suggest that future research should include adaptation and validation of the Health-Related Quality of Life Questionnaire for Nausea and Vomiting of Pregnancy_Portuguese for use in other populations suffering from nausea and vomiting, e.g. cancer patients undergoing chemotherapy. According to Wickham,^29^ the mechanisms of nausea should be highly similar, regardless of its cause.

We also suggest that antiemetic treatment should be individualized and the effect of each intervention should be measured in terms of the patient's own reported outcome. This corroborates the idea that validated instruments should be indicated for investigating the presence, severity and impact of pregnancy nausea and vomiting on the quality of life among patients, who will evaluate for themselves the reported efficacy and effects, and their preferences. It is also important to consider that the impact of nausea and vomiting of pregnancy on quality of life may differ from individual to individual. Some individuals may even suffer continuously, with daily complaints that are secondary to nausea-triggering processes such as first-trimester gestation, a chemotherapy treatment period or a postoperative period. Others may suffer from sporadic nausea, such as symptoms that affect individuals during a trip, for example.

In a study by Dean et al.,^30^ lack of attention towards addressing the symptoms of nausea and vomiting of pregnancy by healthcare professionals was reported. Thus, it is essential to emphasize the importance of using a validated assessment instrument that is available to Brazilian professionals and their patients.

## CONCLUSION

The Health-Related Quality of Life Questionnaire for Nausea and Vomiting of Pregnancy was translated, adapted and validated for the Portuguese language with satisfactory psychometric properties for assessing quality of life, especially with regard to physical health among pregnant women up to their 16^th^ week of gestation, with symptoms of nausea and vomiting. This generated the Health-Related Quality of Life Questionnaire for Nausea and Vomiting of Pregnancy_Portuguese version.

## Annex 1 Health-Related Quality of Life Questionnaire for Nausea and Vomiting of Pregnancy_Portuguese.

**Table:**

Health-Related Quality of Life for Nausea and Vomiting of Pregnancy_Portuguese							
Durante a última semana, quantas vezes você apresentou os seguintes sintomas… (Por favor, marque um círculo na escala para cada sintoma)							
	Nenhuma vez					O tempo todo	
	1	2	3	4	5	6	7
1) Enjoo	□	□	□	□	□	□	□
2) Dor de estômago	□	□	□	□	□	□	□
3) Vômito	□	□	□	□	□	□	□
4) Ânsia de vômito	□	□	□	□	□	□	□
5) Perda de apetite	□	□	□	□	□	□	□
6) Piora dos sintomas no período da tarde	□	□	□	□	□	□	□
7) Não posso comer o quanto desejaria	□	□	□	□	□	□	□
8) Se sente pior quando sente certos cheiros	□	□	□	□	□	□	□
9) Se sente pior quando come certas comidas	□	□	□	□	□	□	□
10) Fadiga	□	□	□	□	□	□	□
11) Se sente esgotada e sem energia	□	□	□	□	□	□	□
12) Se sente exausta	□	□	□	□	□	□	□
13) Se sente cansada	□	□	□	□	□	□	□
14) Se sente emotiva	□	□	□	□	□	□	□
15) Sente menos interesse em sexo	□	□	□	□	□	□	□
16) Se sente para baixo, desanimada, triste, infeliz, deprimida e sem vontade	□	□	□	□	□	□	□
17) Se sente frustrada	□	□	□	□	□	□	□
18) Se sente com raiva por estar enjoada	□	□	□	□	□	□	□
19) Sente que seus sintomas não são de uma gravidez normal	□	□	□	□	□	□	□
20) Sente que você não pode apreciar a gravidez	□	□	□	□	□	□	□
21) Tudo é um esforço	□	□	□	□	□	□	□
22) Sente que você faz menos do que gostaria	□	□	□	□	□	□	□
23) Leva mais tempo para fazer as coisas do que normalmente	□	□	□	□	□	□	□
24) Dificuldade em realizar seu trabalho e atividades	□	□	□	□	□	□	□
25) Dificuldade em manter suas atividades sociais	□	□	□	□	□	□	□
26) Depende do seu parceiro para fazer coisas que você faria normalmente	□	□	□	□	□	□	□
27) Dificuldade em cuidar de sua casa	□	□	□	□	□	□	□
28) Dificuldade em fazer compras	□	□	□	□	□	□	□
29) Dificuldade em cozinhar	□	□	□	□	□	□	□
30) Redução do tempo de trabalho ou outras atividades	□	□	□	□	□	□	□
Se você trabalha fora de casa, você ainda continua trabalhando?	□ Não		□ Sim		□ Não trabalho fora		
Escore: soma das respostas dos itens de 1 a 30, sendo que quanto menor o escore melhor a qualidade de vida.							

This is the Health-Related Quality of Life Questionnaire for Nausea and Vomiting of Pregnancy_Portuguese, which is the validated version for the Portuguese language. How to cite: Piccini A, Tulha AS, Silva SLA, et al. The Brazilian version of the Health-Related Quality of Life Questionnaire for Nausea and Vomiting of Pregnancy: translation, cross-cultural adaptation and reliability – an observational cross-sectional study. Sao Paulo Med J. 2021.
